# Metastatic Cancer Masquerading as Acute Coronary Syndrome

**DOI:** 10.7759/cureus.9628

**Published:** 2020-08-09

**Authors:** Suman Rao, Zachariah Nealy, Alisha Khan, Christopher Nardone

**Affiliations:** 1 Department of Internal Medicine, State University of New York Upstate Medical University, Syracuse, USA; 2 Department of Cardiology, State University of New York Upstate Medical University, Syracuse, USA

**Keywords:** : acute coronary syndrome, cardiac metastasis, metastatic cancer

## Abstract

Patients with heart metastases could present insidiously, with symptoms that mimic those of congestive heart failure or acute coronary syndrome. Our patient initially presented with vague lower sternal and abdominal pain and had a past medical history of coronary artery disease. Her first two troponin levels were elevated, and her EKG was significant for ischemic changes. Echocardiography showed a large mass in the right ventricle and the presence of pericardial effusion. CT scan of the thorax, abdomen, and pelvis showed multiple pulmonary nodules as well as liver metastases. Our patient opted not to pursue further imaging such as cardiac MRI or a liver biopsy. It is imperative that medical professionals are aware of the presentational overlap between acute coronary syndrome and metastatic heart disease, in order to ensure proper diagnosis and management of the latter with echocardiography, cardiac MRI, and possibly surgery.

## Introduction

Although more common than primary cardiac tumors, metastases to the heart are present in roughly 0.7% to 3.5% of the general population at autopsy [1]. They are more common in patients who have known underlying cancers, being present in nearly 9.1% of previously diagnosed cancer patients [2]. Metastatic tumors to the heart can remain asymptomatic, can present with dyspnea on exertion, or can present as chest pain. Echocardiography and cardiac magnetic resonance imaging (CMRI) are modalities that can be helpful in diagnosing heart metastases. Although endomyocardial biopsy remains the gold standard of diagnosis. Difficulties arise when trying to manage a patient with acute coronary symptoms with prior history of coronary artery disease, but no history of cancer.

## Case presentation

We present an 85-year-old female who came into our hospital from an outside facility for further evaluation of subarachnoid hemorrhage and non-ST-segment elevation myocardial infarction (NSTEMI). She initially presented with altered mental status and a mechanical fall. On presentation, she complained of a headache, vague abdominal pain, and pain in the lower sternal area. Further history was significant for anorexia, nausea, vomiting, fatigue, and weight loss that had been going on for the past three to four months.

Her past medical history was significant for coronary artery disease, hypertension, hypothyroidism, iron deficiency anemia, osteoarthritis, and gout. Past surgical history was significant for two cardiac vessel stent placements ten years ago and a right carotid endarterectomy four years ago.

Vital signs on admission were a blood pressure of 151/85 mmHg, heart rate of 92 beats per minute, a temperature of 37 degrees C, respiratory rate of 18 breaths per minute, and oxygen saturation of 95% on room air. On cardiac physical examination, she had no jugular venous distension, and the point of maximal impulse was displaced 2 cm lateral to the midclavicular line. S1 and S2 heart sounds were normal. The rest of the physical examination did not show any abnormalities.

The basic metabolic panel was within normal limits, except for a calcium level of 12.6 mg/dL (normal range of 8.6-10.3 mg/dL). Aspartate aminotransferase (AST) level was 44 units/L (normal range 5-40 units/L) and the alanine aminotransferase (ALT) level was 20 units/L (normal range 5-40 units/L). Ionized calcium was elevated at 1.52 mmol/L (normal range 1.15-1.3 mmol/L). Initial troponin levels were elevated at 0.21 ng/mL (reference range <0.01 ng/dL). The second troponin level measured six hours later was 0.21 ng/mL as well.

**Figure 1:**
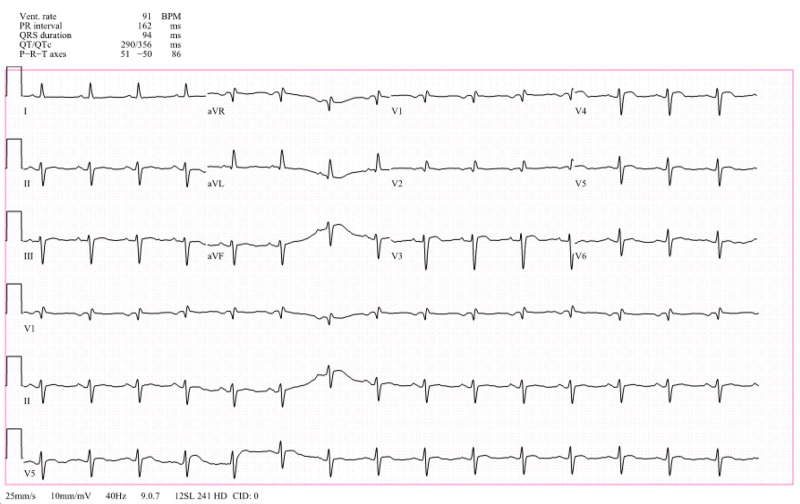
EKG showed sinus rhythm with an incomplete right bundle branch block and a left anterior fascicular block. Anterolateral repolarization alterations present (V3 and V4 especially)

Electrocardiography showed sinus rhythm with an incomplete right bundle branch block and a left anterior fascicular block. Anterolateral repolarization alterations are present (Figure 1).

**Figure 2:**
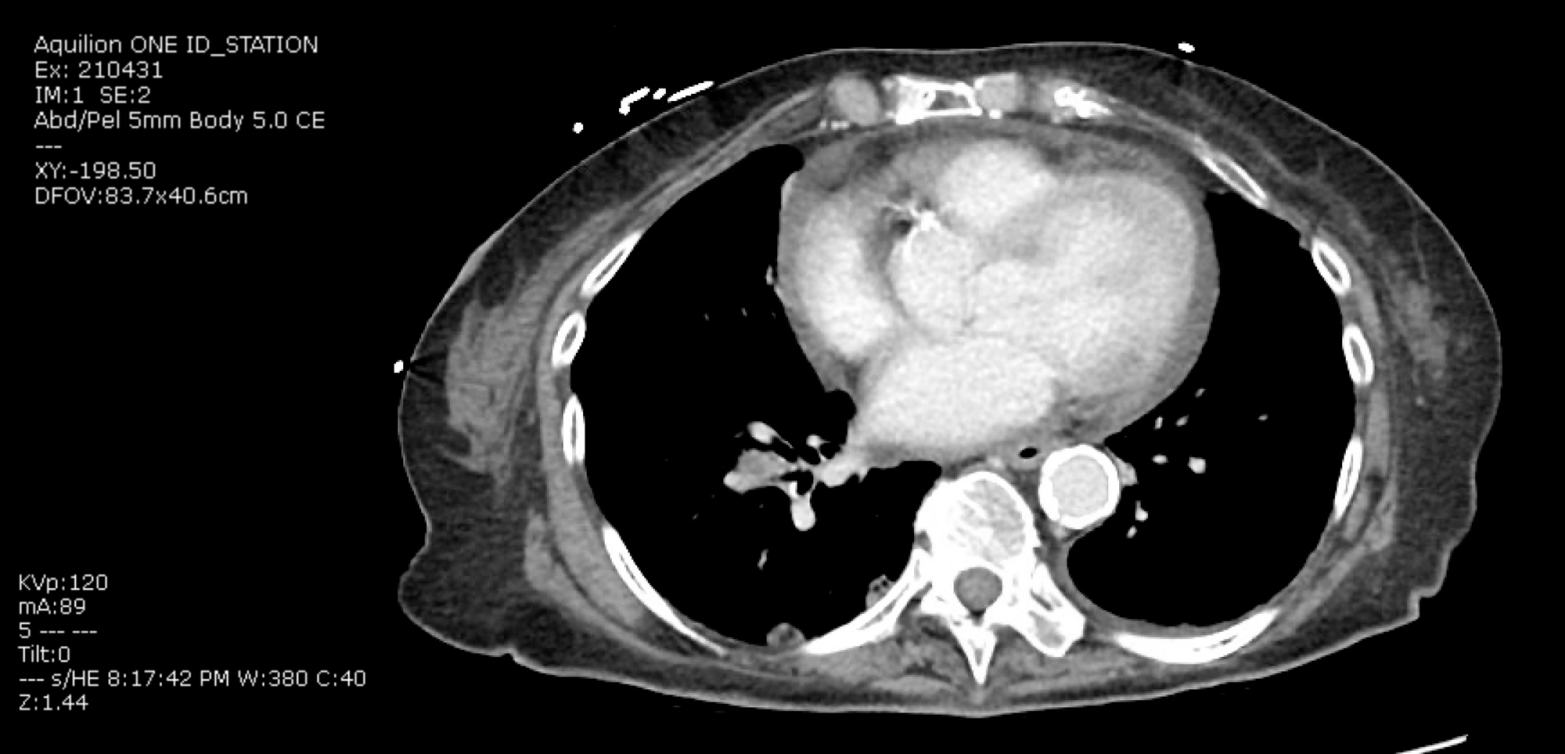
CT Thorax showing moderate pericardial effusion

**Figure 3:**
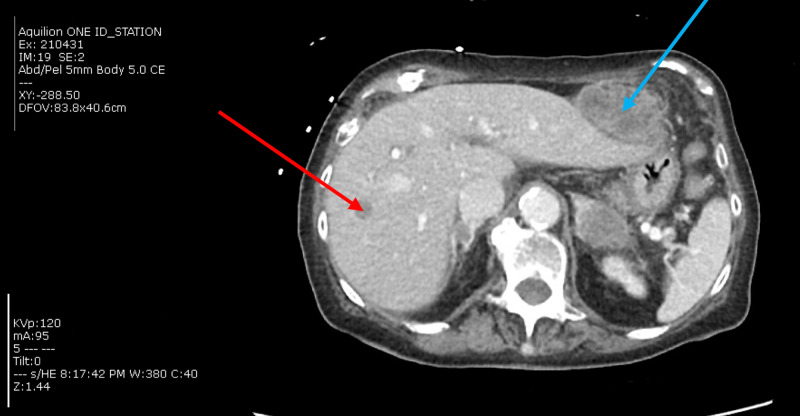
CT abdomen and pelvis shows a large mass (blue arrow) either abutting or arising from in segment 2 of the liver measuring 5.1 cm by 4.0 cm by 6.5 cm with central low attenuation consistent with metastasis. There is another low-attenuation lesion in segment 7 measuring 2.6 cm by 1.2 cm by 1.7 cm (red arrow). Although these may represent simple cysts, metastases are considered more likely

As part of the workup for her fall, a CT thorax was done and demonstrated right lower lobe perihilar mass measuring 2.3 cm by 1.2 cm by 1.2 cm. It also showed multiple pulmonary nodules throughout both lungs, consistent with metastatic disease. The largest index nodule in the left upper lobe measured 1.3 cm in maximum dimension. Moderate sized pericardial effusion was present (Figure 2). CT abdomen and pelvis showed metastatic disease extending throughout the liver, adrenal glands, mesentery, and possibly the pancreas (Figure 3).

**Figure 4:**
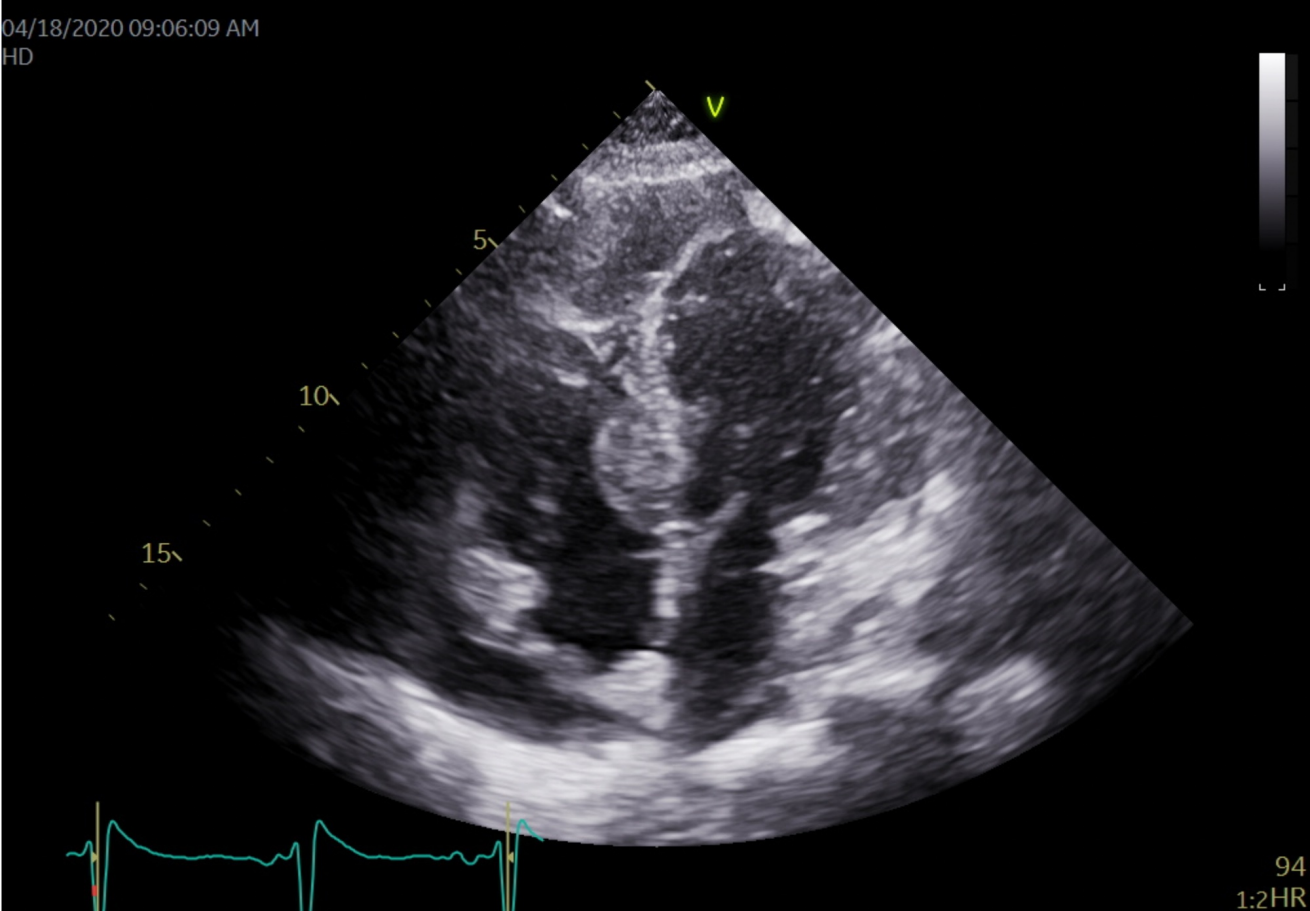
An apical 4 chambers view revealing the pericardial effusion present with a 1.62 cm x 2.45 cm mass in the basal septal portion of the right ventricle

**Figure 5:**
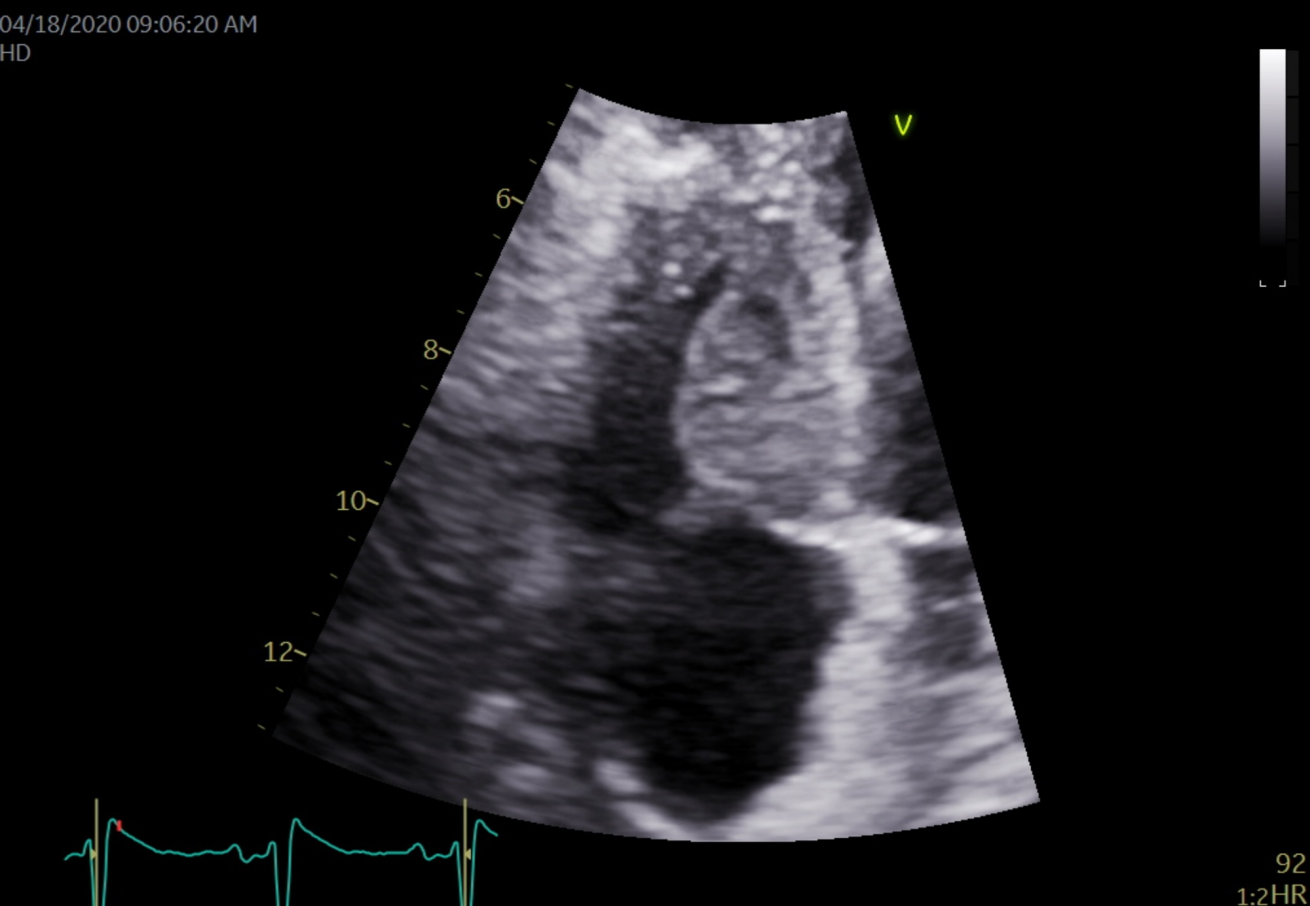
On higher magnification of the mass in the prior image, it is a large 1.62 cm x 2.45 cm mass within the right ventricle that is attached to the septum. The mass appears suspicious for a neoplastic process

**Figure 6:**
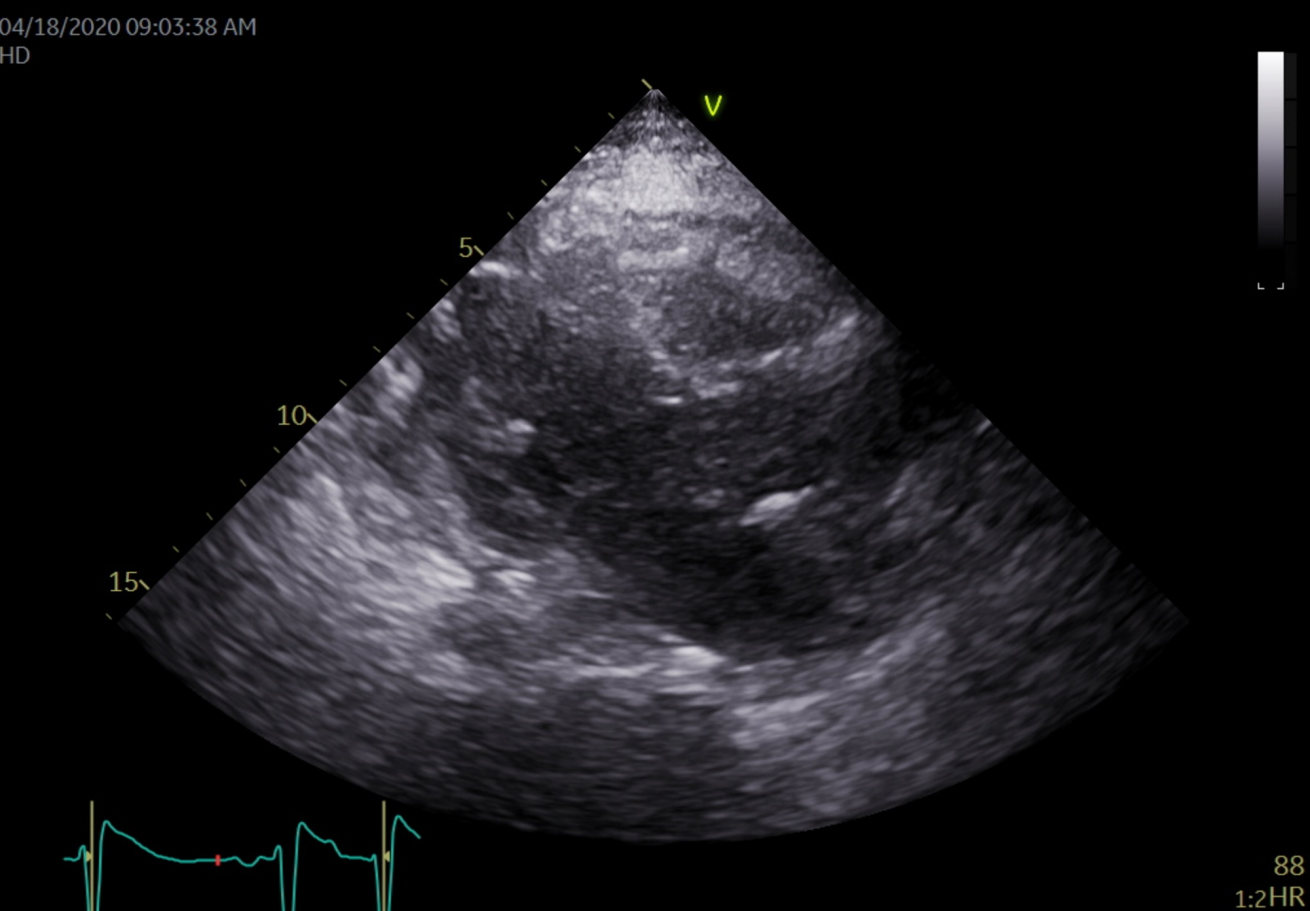
A parasternal long axis view showing the presence of the pericardial effusion that seems to be asymmetrically located closer to the right ventricle. This appears more consistent with malignancy or infection. There was no indication of tamponade physiology present here

**Figure 7:**
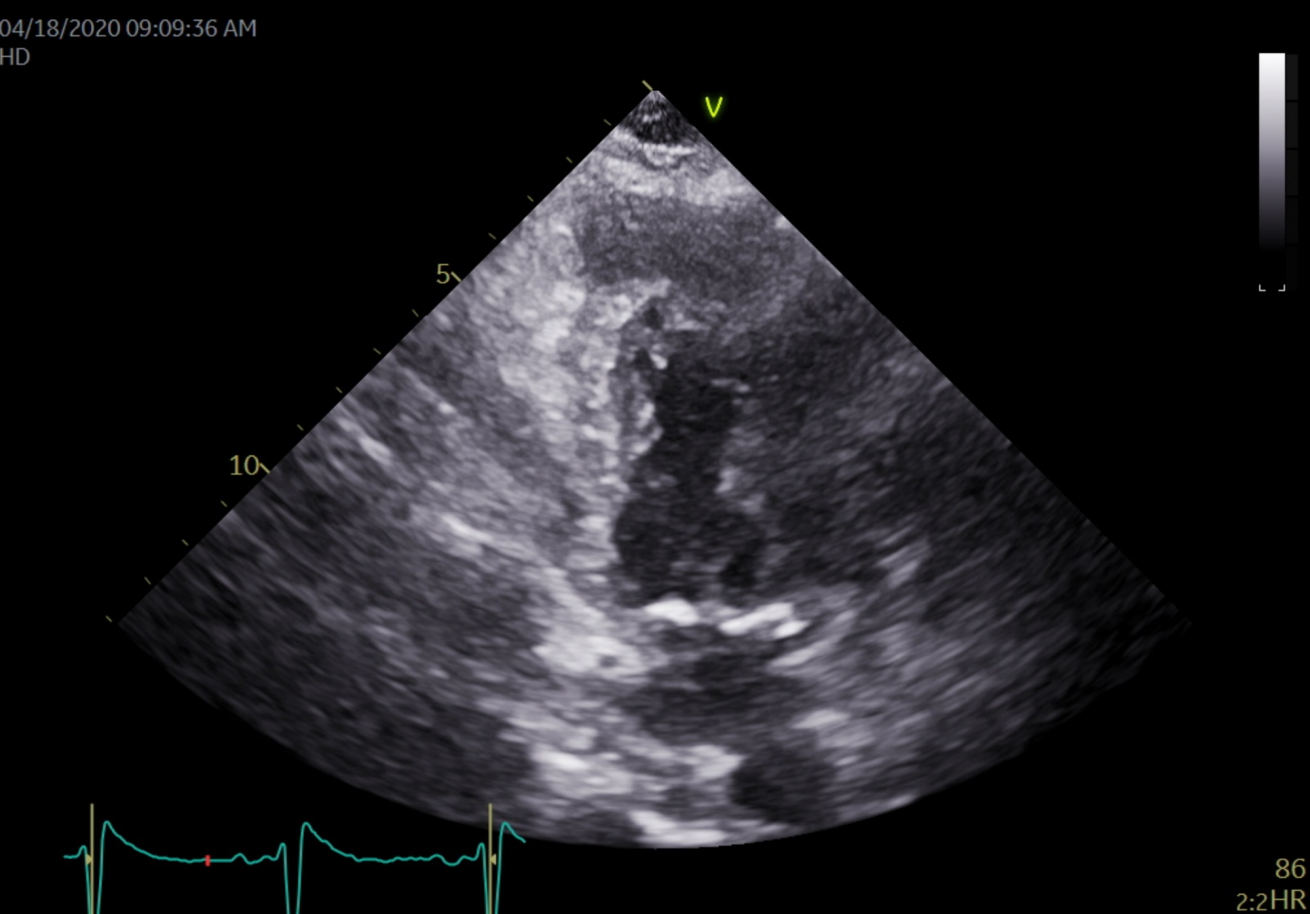
An apical 2-chamber view revealing moderate pericardial effusion is seen without evidence of tamponade

Echocardiography showed a large 1.62 cm by 2.45 cm mass within the right ventricle that is attached to the septum, suspicious for a neoplastic process (Figures 4, 5). The right ventricle and left ventricle systolic function were normal. Impaired left ventricular relaxation and mild aortic valve sclerosis were noted. A moderate pericardial effusion with loculation was also seen (Figures 6, 7). The estimated left ventricular ejection fraction was 60%. No regional wall abnormalities were noted.

After consultation with the cardiology team, the recommendations were to perform a cardiac MRI to further assess the neoplastic process. The options of undergoing an interventional radiologist (IR)-guided liver biopsy and an endobronchial ultrasound (EBUS) were offered to the patient. However, the patient declined to undergo further imaging or interventions and wished to be comfort care. Code status was changed to do not resuscitate/do not intubate (DNR/DNI). Her pain was controlled with oxycodone and morphine. Acutely, her hypercalcemia was managed with normal saline. After medical stabilization, she was referred to hospice care.

## Discussion

The most common primary tumor to metastasize to the heart is lung cancer, comprising nearly 36% of all metastatic heart tumors. Other common tumors that metastasize to the heart are breast cancer, melanomas, lymphomas, some ovarian, and pleural mesothelioma tumors as well [1,3,4].There are several mechanisms by which primary tumors can metastasize to the heart, such as hematological metastasis, contiguous invasion within the thoracic cavity, or extension of the tumor from the inferior vena cava to the right atrium [5].

The clinical manifestations of metastatic heart tumors vary widely with the location of the metastasis. The majority of heart tumors are asymptomatic and are diagnosed during an autopsy. However, metastatic heart tumors can cause dyspnea on exertion, arrhythmias, and symptoms of heart failure [6].

Our patient presented with myocardial and pericardial involvement of the metastases. We believe that the pericardial metastatic involvement is the cause for moderate pericardial effusion with loculation seen on echocardiography. This is not a usual finding in metastatic heart disease. Myocardial metastatic involvement could lead to a multitude of issues in theory, including arrhythmias, such as atrial/ventricular fibrillation. It could also mimic several laboratory and electrocardiography findings that are present in acute coronary syndrome.

Physical examination in patients with metastatic tumors to the heart may be significant for newly found murmurs secondary to valvular dysfunction or rubs due to pericardial abnormalities. Electrocardiography could show ST-segment variation, atrial/ventricular arrhythmias, or other localized conduction abnormalities. Echocardiography is warranted as the next step in diagnosis. Echocardiography demonstrates the location and size of metastatic lesions within the mediastinum. It also helps visualize the presence of pericardial effusions [7]. However, cardiac magnetic resonance imaging (CMR) is necessary to overcome some limitations of echocardiography [8]. CMR allows for higher resolution images, identification of intramyocardial masses, and visualization of extracardiac structures [9]. Regardless of the imaging modalities used, tissue histopathology is the confirmatory measure to diagnose metastatic heart disease, and can be carried out by endomyocardial biopsy, open biopsy through a thoracotomy, or pericardiocentesis with cytology [1].

The treatment of cardiac metastases is based on the management of the presenting symptoms. For patients presenting with arrhythmias secondary to metastases, antiarrhythmics, and radiofrequency ablation could temporarily help resolve the problem. Patients presenting with symptoms of cardiac tamponade and hemodynamic instability would need a pericardiocentesis or a pericardial window. However, more likely than not, patients with metastatic heart disease will have a great tumor burden requiring palliative service to facilitate discussion with patients and families about prognosis. Surgical resection of metastases to the heart is indicated in patients who not only show symptoms of intracardiac obstruction but also have a favorable prognosis. Some types of metastatic tumors respond to radiotherapy and chemotherapy as well [10].

Metastatic heart disease mimicking acute coronary syndrome

Invasion and metastasis of the tumor into the myocardium of the heart could lead to several findings that are indicative of acute coronary syndrome. Myocardial metastasis could lead to increased levels of troponin, and ST-segment abnormalities, as seen in our patient. Patients may also exhibit symptoms of chest pain, typical of the acute coronary syndrome. Our patient had vague abdominal pain on presentation, elevated troponins, ST-segment changes, and a history of coronary artery disease. However, in this case, echocardiography was necessary to determine the etiology of her current presentation, which was metastatic cancer. It is important for medical professionals to suspect metastasis to the heart as a cause of typical chest pain in patients with a history of cancer, so as not to incorrectly pursue the management of coronary artery disease. Our patient did have elevated troponin; however, it was the same value for all three lab draws, insinuating that the elevated cardiac markers were secondary to metastases, as opposed to active ischemia/infarction. In these patients, echocardiography and cardiac MRI would be warranted as opposed to keeping a low threshold for coronary angiography or other cardiovascular imaging modalities.

## Conclusions

Metastatic disease to the heart is a relatively rare condition that usually presents in patients with highly advanced cancer. Tumors that metastasize to the myocardium of the heart could lead to typical chest pain symptoms, elevated cardiac biomarkers, and ST-segment changes on EKG. In patients with a previous history of coronary artery disease, it could be difficult to differentiate between acute coronary syndrome and metastatic heart disease on presentation. These patients would benefit from echocardiography and further imaging modalities such as cardiac MRI in order to aid in further management.
